# Presence of Antinuclear Antibodies in the Cerebrospinal Fluid and in the Serum in a Patient With Sjögren’s Syndrome and Systemic Lupus: A Case Report

**DOI:** 10.1155/crii/3015090

**Published:** 2026-09-21

**Authors:** Cherqaoui Ayman, Taif Hiba, Laraki Kenza, Fadili Hajar, Snoussi Malak, Zrara Abdelhamid, El Bakkouri Jalila

**Affiliations:** ^1^ Mohammed VI University of Sciences and Health—Faculty of Pharmacy, Casablanca, Morocco; ^2^ Cheikh Khalifa International University Hospital—Medical Laboratory, Mohammed VI University of Sciences and Health, Casablanca, Morocco; ^3^ Mohammed VI University of Sciences and Health—Faculty of Medicine, Casablanca, Morocco; ^4^ Mohammed VI National Laboratory of Medical Analysis, Casablanca, Morocco; ^5^ Immunopathology-Immunotherapy-Immunomonitoring Laboratory, Mohammed VI University of Sciences and Health, Casablanca, Morocco

**Keywords:** antinuclear antibodies, case report, cerebrospinal fluid, neuropsychiatric lupus, Sjögren syndrome, systemic lupus erythematosus

## Abstract

Systemic lupus erythematosus (SLE) may overlap with Sjögren’s syndrome (SS), leading to complex systemic involvement, including neurological and pulmonary manifestations. We report a 60‐year‐old woman with a 10‐year history of SLE–SS overlap presenting with headaches and bilateral visual impairment. Hydroxychloroquine (HCQ) had been discontinued 4 months prior due to HCQ‐related optic neuropathy confirmed on optical coherence tomography (OCT). Ophthalmologic evaluation revealed bilateral optic neuritis. Immunological testing showed high‐titre antinuclear antibodies (ANAs) in both serum (1:640) and cerebrospinal fluid (CSF) (1:320), with positive anti‐dsDNA, anti‐SSA/Ro52, anti‐Sm/RNP, and anti‐ribosomal P antibodies. CSF analysis was normal, without pleocytosis, hyperproteinorrhachia, oligoclonal bands, or intrathecal IgG synthesis (IgG index 0.61). Brain MRI showed non‐enhancing periventricular demyelinating lesions, and thoracic CT revealed bilateral bronchiectasis. The patient received intravenous methylprednisolone (500 mg/day × 3 days) followed by oral corticosteroids, with leflunomide added as an immunomodulatory agent given the overlap syndrome context, resulting in a favourable clinical outcome. The absence of intrathecal immunoglobulin synthesis and oligoclonal bands supports blood–brain barrier dysfunction rather than a primary demyelinating process, highlighting the diagnostic challenge in SLE–SS overlap. ANA detection in CSF may serve as a supportive marker for neuropsychiatric lupus (NPSLE) in overlap syndromes.

## 1. Introduction

Systemic lupus erythematosus (SLE) is a systemic autoimmune disease characterised by a wide spectrum of clinical manifestations resulting from multi‐organ involvement. Sjögren’s syndrome (SS), also a systemic autoimmune disease, may be associated with SLE in the context of overlap syndromes, reflecting increased pathophysiological complexity [1]. Central nervous system (CNS) involvement in SLE represents a major diagnostic challenge, particularly when presenting as optic neuritis or demyelinating lesions, which may mimic conditions such as multiple sclerosis (MS) or neuromyelitis optica spectrum disorder (NMOSD) [2].

The distinction between primary demyelinating disease and neuropsychiatric lupus (NPSLE) is therapeutically critical. Normal cerebrospinal fluid (CSF) parameters—including the absence of oligoclonal bands and the lack of intrathecal immunoglobulin synthesis—argue against a primary demyelinating process and support a systemic autoimmune aetiology. In this context, detection of antinuclear antibody (ANA) in CSF has been proposed as an additional immunological marker of CNS involvement in NPSLE [3].

Pulmonary involvement associated with SS frequently manifests as inflammatory and structural abnormalities, including bronchiectasis, pulmonary cysts, and small airway disease [4]. The coexistence of autoimmune neurological involvement and structural pulmonary disease within an SLE–SS overlap syndrome raises complex diagnostic, pathophysiological, and therapeutic challenges, which we address in this case report.

## 2. Patient and Observation

### 2.1. Patient Information

We report the case of a 60‐year‐old woman with a 10‐year history of SLE associated with SS, complicated by neurological and pulmonary involvement. Initial manifestations included diffuse inflammatory polyarthralgia, progressive alopecia, erythematous lesions on the arms, and sicca syndrome (oral and ocular dryness). The family history was notable for first‐degree consanguinity and multiple early deaths in siblings. She had four spontaneous abortions at 4 months of gestation and had undergone umbilical hernia repair 15 years prior.

Regarding background therapy: the patient had been receiving hydroxychloroquine (HCQ, Plaquenil 400 mg/day) since SLE diagnosis, in addition to pilocarpine (Salagene 10 mg/day) for sicca symptoms and low‐dose oral prednisolone titrated according to disease activity. HCQ was discontinued approximately 4 months before the current episode following detection of optic neuropathy on optical coherence tomography (OCT), consistent with HCQ‐related ocular toxicity. Pneumococcal vaccination (Pneumovax 23) was administered in April 2025.

### 2.2. Timeline of Current Episode

A detailed chronological summary of the patient’s clinical course, including all investigational findings, treatments, and outcomes, is presented in Table 1.

**Table 1:** Timeline of clinical events, investigational findings and management in the reported patient.

Time point	Clinical events, findings and management
2015	Diagnosis of SLE associated with Sjögren’s syndrome (SS). Background therapy initiated including hydroxychloroquine (HCQ, Plaquenil 400 mg/day) and symptomatic treatment for sicca syndrome (pilocarpine, Salagene 10 mg/day).
2015–2024 (chronic course)	Ongoing chronic symptoms: diffuse inflammatory polyarthralgia, progressive alopecia, erythematous cutaneous lesions on the arms, sicca syndrome (oral and ocular dryness). Low‐dose oral prednisolone (Cortancyl) titrated according to disease activity.
~4 months before current admission	Hydroxychloroquine discontinued following detection of optic neuropathy on optical coherence tomography (OCT), consistent with HCQ‐related ocular toxicity.
December 2024	New onset of exertional dyspnoea. Thoracic CT: bilateral cystic bronchial dilatation (bronchiectasis). Bilateral crackles on auscultation.
January 2025	Immunological work‐up: ANA positive, speckled pattern 1:640; RF and anti‐CCP negative; proteinuria 0.12 g/24 h.
February 2025	Extended antibody panel: anti‐dsDNA positive, anti‐ribosomal P positive, anti‐SSA/Ro52 positive, anti‐Sm/RNP strongly positive. Normal immunodeficiency panel.
March 2025	Respiratory exacerbation (purulent sputum): antibiotic therapy (amoxicillin‐clavulanate).
April 2025	Pulmonary function tests (PFTs): small airway obstruction, moderate DLCO reduction (48%), notable improvement after beta‐2 agonists. Oral prednisolone adjusted (Cortancyl 5 mg/day + KCl + calcifix).
June–July 2025	Polyarticular flare (hands, wrists, elbows). C3 consumed; CRP 10.68 mg/L. Prednisolone increased to 7.5 mg/day. Leflunomide (Arava, 20 mg/day) introduced for immunomodulation. Ophthalmologic review: no diabetic retinopathy, no lupus retinopathy.
September 2025 (current admission)	Hospital admission for neurological and pulmonary management. Vital signs: BP 130/82 mmHg/HR 83 bpm, SpO_2_ 92% on room air, apyrexial. Clinical examination: bilateral crackles, mild balance disturbance, polyarthralgia of small hand joints, diffuse hyperpigmentation, no active skin lesions, no cranial nerve deficit. Lumbar puncture: normal cellularity and protein levels; ANA positive in CSF 1:320 (speckled); IgG index 0.61; no oligoclonal bands. Brain MRI: periventricular white matter demyelinating lesions, no gadolinium enhancement. Spinal MRI: no signal abnormality. Orbital MRI: bilateral optic neuritis with optic nerve sheath dilation. IV methylprednisolone (Solumedrol) 500 mg/day × 3 days; then oral relay at 30 mg/day. Enoxaparin (Lovenox) 0.4 mg SC once daily throughout bolus phase.
Follow‐up (~6 months)	Favourable clinical evolution: complete respiratory improvement, stabilisation of neurological manifestations, partial regression of arthralgia. No ocular relapse on ophthalmologic follow‐up. No retinopathy. Liver function tests normal under leflunomide. Progressive corticosteroid tapering to 7.5 mg/day maintenance.

## 3. Diagnostic Assessment

### 3.1. Immunological and Biological Investigations

A comprehensive immunological work‐up was performed. Results are summarised in Table 2.

**Table 2:** Results of biological and immunological investigations performed.

Parameter	Result
Antinuclear antibodies (ANA), serum	Positive, speckled pattern 1:640
ANA, CSF	Positive, speckled pattern 1:320
Anti‐double‐stranded DNA (anti‐dsDNA)	Positive
Anti‐ribosomal P	Positive
Anti‐SSA/Ro52	Positive
Anti‐Sm/RNP	Strongly positive
Anti‐SSB	Negative
Rheumatoid factor	Negative
Anti‐CCP	Negative
Complement C3	Normal (0.97 g/L)
Complement C4	Low/consumed (0.097 g/L)
C‐reactive protein (CRP)	Normal (4.4 mg/L)
Serum protein electrophoresis	Hypoalbuminemia (36.4 g/L) with increased alpha‐1 globulins; moderate hypogammaglobulinemia (7.6 g/L)
24‐h proteinuria	0.12 g/24 h (non‐significant)
Cryoglobulinemia	Negative
Antiphospholipid antibodies: Anti‐cardiolipin IgG Anti‐cardiolipin IgM Anti‐β2‐glycoprotein I IgG Anti‐β2‐glycoprotein I IgM Lupus anticoagulant	Negative Positive Negative Positive Not detected
IgG index (CSF)	0.61 (no intrathecal IgG synthesis)
Oligoclonal bands (CSF)	Absent (isoelectric focusing gel)
CSF cellularity and protein	Normal—no pleocytosis, no hyperproteinorrhachia

Given the family history of first‐degree consanguinity and multiple early deaths among siblings (§2.1), a primary immunodeficiency work‐up was also performed before fully attributing the phenotype to the SLE–SS overlap. This included lymphocyte subpopulation analysis and serum immunoglobulin levels with subclasses, both of which were normal, together with complement fractions C3 and C4 (Table 2). Total complement haemolytic activity (CH50) was not measured in this patient; we acknowledge this as a limitation of the immunodeficiency work‐up and recommend its inclusion, alongside individual classical‐pathway component levels, in similar presentations to further exclude a hereditary complement deficiency.

### 3.2. CSF Analysis

**Figure 1:**
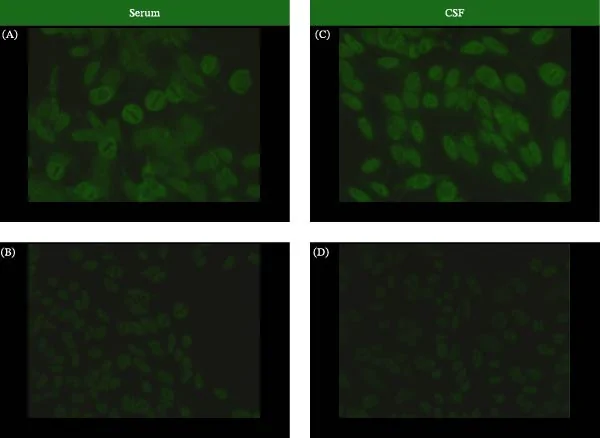
Detection of antinuclear antibodies (ANA) by indirect immunofluorescence on HEp‐2 cells showing a speckled pattern: (A, B) in serum (titre 1:640); (C, D) in the cerebrospinal fluid (CSF) of our patient (titre 1:320). The lower fluorescence intensity in CSF panels compared to serum is consistent with passive antibody transfer across the blood–brain barrier rather than intrathecal synthesis. The subfigures (A and B) (serum) and subfigures (C and D) (CSF) each show two independent microscopic fields from the same slide and specimen preparation, acquired during the same session, and do not represent different time points, dilutions, or magnifications.

Lumbar puncture revealed normal CSF cellularity and protein levels (no pleocytosis and no hyperproteinorrhachia). The IgG index was 0.61—below the threshold for intrathecal IgG synthesis—and oligoclonal bands were absent on the isoelectric focusing gel. Indirect immunofluorescence on HEp‐2 cells demonstrated positive ANA in CSF with a speckled pattern at a titre of 1:320 (Figure 1). The absence of intrathecal synthesis indicates that CSF ANA resulted from passive transfer across a dysfunctional blood–brain barrier rather than local intrathecal production.

Serum ANA titre was determined by indirect immunofluorescence on the HEp‐2 cell substrate (Kallestad HEp‐2 kit), using the laboratory’s standard screening dilution of 1:160 followed by two‐fold serial dilution to the endpoint. CSF was tested undiluted, owing to the expected low baseline immunoglobulin concentration in this compartment; the reported CSF titre (1:320) was estimated semi‐quantitatively by comparison of fluorescence intensity with the serum reference scale, rather than by an independent serial dilution series performed on the CSF specimen itself. Our laboratory’s written protocol formally validates undiluted CSF testing for anti‐NMO, anti‐MOG and anti‐NMDA‐receptor antibodies but does not extend this validation to ANA; the CSF ANA result reported here therefore represents an extension of the serum methodology to a specimen type for which matrix‐specific validation is lacking, an acknowledged limitation given that most autoantibody assays are not standardised for CSF use.

Anti‐aquaporin‐4 (AQP4) antibody testing was not available at the time of this evaluation and constitutes an acknowledged limitation. AQP4‐IgG positivity is highly specific for NMOSD and, if present, would have directly modified the diagnostic conclusion and therapeutic strategy. We therefore recommend that anti‐AQP4 and anti‐MOG antibodies be systematically tested in any similar presentation [5]. The absence of oligoclonal bands in this case is consistent with published observations in neuropsychiatric SLE rather than primary demyelinating disease [6].

### 3.3. Imaging

Brain MRI revealed periventricular demyelinating lesions in the white matter without gadolinium enhancement—a feature arguing against active inflammatory demyelination as seen in relapsing‐remitting MS and supporting a vasculitic or autoimmune‐mediated mechanism. Spinal MRI showed no signal abnormalities, ruling out spinal cord involvement (which would be expected in NMOSD). Orbital MRI demonstrated bilateral optic neuritis with dilation of the optic nerve sheaths. Thoracic CT revealed bilateral cystic bronchial dilatation (bronchiectasis) without mediastinal lymphadenopathy. PFTs showed small airway obstruction with moderate DLCO reduction (48%), with notable improvement after beta‐2 agonists.

### 3.4. Diagnosis

SLE associated with SS with pulmonary involvement (bilateral bronchiectasis, small airway obstruction) and neuropsychiatric involvement (bilateral optic neuritis, periventricular white matter lesions). The neurological presentation was attributed to neuropsychiatric SLE on the basis of the clinical context, the immunological profile (multiple SLE‐related autoantibodies in both serum and CSF), the absence of intrathecal IgG synthesis, the absence of oligoclonal bands, and the absence of spinal cord lesions.

## 4. Therapeutic Interventions

### 4.1. Rationale for Treatment Choices

The treatment approach was guided by the coexistence of SLE and SS with active neuropsychiatric and pulmonary involvement, the presence of antiphospholipid antibodies (IgM), and the prior discontinuation of HCQ due to documented ocular toxicity.

#### 4.1.1. Corticosteroids

Given the vision‐threatening presentation (bilateral optic neuritis), the patient initially received IV methylprednisolone (Solumedrol) 500 mg/day for 3 days during hospitalisation, followed by oral relay at 30 mg/day, subsequently tapered to 10 mg/day then 7.5 mg/day as maintenance. High‐dose IV corticosteroids are the cornerstone of initial NPSLE management for vision‐threatening or severe neurological manifestations.

#### 4.1.2. Leflunomide

Leflunomide was introduced as an immunomodulatory agent for several reasons. Its active metabolite (teriflunomide) inhibits dihydroorotate dehydrogenase, blocking de novo pyrimidine synthesis and suppressing activated T and B lymphocyte proliferation [5]. Although not a first‐line agent in standard SLE or SS guidelines, leflunomide has demonstrated clinical efficacy in primary SS for extraglandular systemic manifestations, with documented T‐cell regulation [7]. In the SLE context, it has been used off‐label for refractory vasculitis and neuropathies. It was chosen to complement corticosteroids in controlling autoimmune activity across both SLE and SS. A recognised adverse effect to monitor is leflunomide‐induced subacute cutaneous lupus [5].

#### 4.1.3. HCQ

HCQ is recommended as background therapy in both SLE and SS, providing immunomodulatory, cardiovascular, and anti‐thrombotic benefits. It was discontinued due to documented optic neuropathy on the OCT. Follow‐up ophthalmologic examination (28/07/2025) showed no retinopathy, and visual symptoms improved after HCQ cessation. Re‐introduction at a reduced dose (≤5 mg/kg/day) under close ophthalmological surveillance should be considered once optic neuritis has fully resolved.

A structured overview of all pharmacological interventions, dosages, indications, adverse effects, and targeted manifestations is provided in Table 3.

**Table 3:** Summary of pharmacological management—dosages, indications, adverse effects and targeted manifestations.

Medication	Dosage and duration	Indication/target	Adverse effects and monitoring	SLE/SS manifestations targeted
Corticosteroids (prednisolone/ Cortancyl)	IV methylprednisolone 500 mg/day × 3 days (bolus); oral relay 30 mg/day, tapered to 10 mg/day then 7.5 mg/day (maintenance). Prior chronic low‐dose 5–10 mg/day.	NPSLE (bilateral optic neuritis, periventricular lesions); articular flare; pulmonary inflammation	Hyperglycaemia (HbA1c monitored, 5.7%); osteoporosis (Vit D + Ca^2+^); hypokalaemia (KCl); thromboembolic risk (LMWH during bolus phase); infection screening	SLE: neuropsychiatric, articular, cutaneous SS: systemic
Leflunomide (Arava)	20 mg/day (one tablet/day), ongoing	Immunomodulation of extraglandular SS manifestations; articular involvement refractory to low‐dose steroids; complement corticosteroids in SLE–SS overlap	Hepatotoxicity (LFTs normal throughout: AST 30, ALT 15 UI/L); teratogenicity; leflunomide‐induced subacute cutaneous lupus (rare—monitor skin)	SS: extraglandular/systemic SLE: articular (off‐label), autoimmune control
Hydroxychloroquine (Plaquenil) (Suspended)	400 mg/day (prior long‐term therapy). Discontinued ~4 months before admission due to OCT‐confirmed HCQ optic neuropathy. Re‐introduction at ≤5 mg/kg/day to be discussed after optic neuritis resolution.	Standard background SLE/SS therapy; anti‐thrombotic; anti‐inflammatory; metabolic benefit	Retinal toxicity (OCT confirmed at discontinuation; repeat OCT 28/07/2025: no retinopathy). Re‐introduction requires ophthalmology clearance.	SLE and SS: background immunomodulation, anti‐thrombotic
Pilocarpine (Salagene)	10 mg/day, ongoing	Sicca syndrome (xerostomia, xerophthalmia) associated with SS	Cholinergic side effects (sweating, increased salivation); not immunomodulatory	SS: sicca symptoms
Beta‐2 agonists (bronchodilators)	As needed (inhaled)	Small airway obstruction (DLCO 48%); bilateral bronchiectasis	Well tolerated; notable PFT improvement after administration	SS: pulmonary—bronchiectasis, airway obstruction
LMWH (enoxaparin 0.4 mg SC)	Once daily SC, throughout bolus corticosteroid phase, then discontinued	Thromboembolic prophylaxis during high‐dose corticosteroids; antiphospholipid antibodies positive (IgM)	Bleeding risk (standard monitoring); limited duration	Thromboprophylaxis (SLE/APS context)
Vit D + calcium + potassium supplementation	Standard preventive doses, ongoing during corticosteroid therapy	Prevention of corticosteroid‐induced osteoporosis and hypokalaemia	Minimal; electrolyte and bone density monitoring	Corticosteroid side‐effect prevention

### 4.2. Supportive and Prophylactic Measures

Thromboembolic prophylaxis with LMWH (enoxaparin 0.4 mg SC once daily) was administered throughout the bolus corticosteroid phase, given antiphospholipid antibody positivity. Supplementation with vitamin D, calcium and potassium was initiated to prevent corticosteroid‐related complications. Bronchodilators were prescribed for airway obstruction and antibiotic therapy was given during respiratory exacerbations.

## 5. Follow‐Up and Outcome

The follow‐up period extended approximately 6 months from hospitalisation to manuscript submission. The clinical course was favourable: complete improvement in respiratory function was observed, with stabilisation of neurological manifestations (no new demyelinating lesions and no optic relapse on ophthalmologic follow‐up). Arthralgia improved partially. Liver function tests remained normal throughout leflunomide therapy. No leflunomide‐induced cutaneous lupus was observed. Progressive corticosteroid tapering was achieved.

## 6. Discussion

### 6.1. Neuropsychiatric Manifestations in SLE–SS Overlap

SLE is a systemic autoimmune disease with neuropsychiatric manifestations grouped under the general term NPSLE, constituting one of the most severe visceral complications [1]. In our patient, the presence of ANA, anti‐dsDNA and anti‐ribosomal P antibodies in the CSF—in the clinical context of headaches, bilateral optic neuritis and periventricular white matter lesions—supports the diagnosis of NPSLE [2]. Anti‐ribosomal P antibodies have been particularly associated with neuropsychiatric manifestations of SLE, further supporting this attribution [2].

### 6.2. Rationale for Emphasising ANA in CSF

Among the immunological markers detected, ANA was the only autoantibody for which a directly paired serum/CSF titre was available at the same time point, allowing comparison between intrathecal and systemic immune compartments. Detection of CSF ANA at 1:320—in the absence of intrathecal IgG synthesis (IgG index 0.61) and oligoclonal bands—indicates passive antibody transfer across a dysfunctional blood–brain barrier rather than local intrathecal production. We do not propose CSF ANA as a validated, stand‐alone diagnostic marker of NPSLE: paired serum/CSF studies have shown that CSF ANA, like several other autoantibodies, does not reliably distinguish central NPSLE from non‐NPSLE SLE or even from non‐lupus causes of blood–brain barrier breach such as septic meningitis and would plausibly also be detectable in SLE patients without clinical NPSLE [8]. Anti‐ribosomal P and anti‐SSA/Ro antibodies have shown stronger, syndrome‐specific associations with NPSLE in larger cohorts [9, 10] and, being reported as concentration‐type (U/mL) results, offer a more quantitative readout than ANA titration by immunofluorescence. In the present case, CSF ANA is therefore best interpreted as supportive evidence of blood–brain barrier dysfunction in a patient with a clinical presentation already strongly suggestive of NPSLE rather than as an independent diagnostic marker.

#### 6.2.1. Pitfalls in CSF Autoantibody Interpretation: Broader Literature

The interpretation of CSF autoantibody findings in atypical presentations warrants caution regarding several potential pitfalls. Fragoso‐Loyo et al. [8] found that CSF ANA, anti‐dsDNA, anti‐cardiolipin and anti‐ribosomal P antibodies did not distinguish central NPSLE from non‐NPSLE SLE in a paired serum/CSF cohort and that all antibodies tested were similarly elevated in SLE patients with septic meningitis, reflecting non‐specific blood–brain barrier breach rather than NPSLE‐specific pathology; only anti‐NMDA‐receptor antibody discriminated central NPSLE in that study. Using a human proteome microarray, Hu et al. [9] similarly found that generic ANA did not discriminate NPSLE, whereas anti‐ribosomal P proteins and anti‐SSA/Ro were significantly more frequent in NPSLE than in non‐NPSLE SLE or non‐SLE controls. In a systematic review of over 8,000 patients, Sciascia et al. [10] concluded that no single autoantibody is reliably associated with NPSLE as a whole; only antiphospholipid antibodies (mainly lupus anticoagulant, associated with cerebrovascular events) and anti‐ribosomal P antibodies (associated with psychosis) reached syndrome‐specific significance. West et al. [11], in an early prospective study, similarly highlighted the limited and manifestation‐specific diagnostic value of serum and CSF testing in NPSLE. Taken together, these data indicate that elevated CSF autoantibody levels—including ANA—should not be over‐interpreted as NPSLE‐specific in isolation and are best considered alongside clinical, imaging and CSF inflammatory parameters, as in the present case.

### 6.3. Distinguishing NPSLE From Primary Demyelinating Disease

The differential diagnosis between NPSLE and primary demyelinating disease—particularly MS and NMOSD—is therapeutically critical. Several features in this case support NPSLE. First, the CSF profile was entirely normal: no pleocytosis, no hyperproteinorrhachia, no oligoclonal bands and a normal IgG index—features inconsistent with MS or NMOSD [6]. Second, periventricular white matter lesions showed no gadolinium enhancement, arguing against active inflammatory demyelination. Third, spinal MRI was normal, whereas NMOSD characteristically involves longitudinally extensive transverse myelitis. Fourth, the patient carries multiple SLE‐relevant autoantibodies, providing a compelling systemic autoimmune context.

We acknowledge that anti‐AQP4 antibody testing was not performed—an important limitation. AQP4‐IgG positivity is highly specific for NMOSD and would have directly altered the diagnostic conclusion and management. Anti‐AQP4 and anti‐MOG antibodies should be tested in all similar presentations [5]. The focus on NMOSD rather than MS as the primary differential was motivated by the bilateral nature of the optic neuritis and the absence of oligoclonal bands—features more characteristic of NMOSD than MS in the autoimmune overlap context [3].

### 6.4. Pulmonary Involvement

Pulmonary involvement is a well‐established complication of SS, with a spectrum ranging from pleurisy to interstitial lung disease and bronchiectasis, as observed in our case [12]. Bilateral bronchiectasis is the correct radiological term for cystic bronchial dilatation found on CT. These manifestations may be underdiagnosed but are prognostically significant, requiring evaluation with thoracic CT and PFTs [12].

### 6.5. Treatment Considerations

The therapeutic challenge arose from the need to simultaneously address NPSLE, SS‐related pulmonary disease, articular flare and prior HCQ discontinuation. High‐dose IV corticosteroids are the cornerstone of initial NPSLE management for vision‐threatening manifestations. The addition of leflunomide provided complementary immunomodulation targeting extraglandular SS manifestations and articular SLE involvement refractory to low‐dose corticosteroids [5, 7]. Rituximab, a B‐cell depleting agent increasingly used in refractory NPSLE, was not considered by the treating clinicians in this case, as the combination of high‐dose corticosteroids and leflunomide achieved a favourable response across all affected organ systems; it remains a relevant option should relapse or refractory disease occur.

The temporary absence of HCQ deserves emphasis: HCQ is recommended as background therapy in both SLE and SS, providing immunomodulatory, cardiovascular, and anti‐thrombotic benefits. Discontinuation was driven by documented HCQ‐related optic neuropathy. Given the subsequent visual improvement and normal follow‐up OCT, re‐evaluation for HCQ re‐introduction at a lower dose under close ophthalmological surveillance should be prioritised at follow‐up.

## 7. Conclusion

This case illustrates the diagnostic and therapeutic complexity of SLE–SS overlap syndrome with concurrent neuropsychiatric and pulmonary manifestations. The combination of normal CSF parameters (absence of oligoclonal bands and normal IgG index) with positive CSF ANA supports blood–brain barrier dysfunction rather than a primary demyelinating disease. Anti‐AQP4 and anti‐MOG antibody testing is recommended in all similar presentations to exclude NMOSD. High‐dose IV corticosteroids followed by leflunomide provided effective disease control across multiple organ compartments. The role of HCQ as background therapy requires systematic re‐evaluation, balancing systemic benefits against ophthalmological risk. Further prospective studies are needed to clarify the optimal immunomodulatory strategy for NPSLE in the SLE–SS overlap.

## Author Contributions

Cherqaoui Ayman contributed to the conception of the case report, data acquisition and analysis and draughting of the manuscript. Taif Hiba and Laraki Kenza contributed to the data acquisition and analysis of laboratory findings. Fadili Hajar and Snoussi Malak contributed to data interpretation and critical revision of the manuscript. Zrara Abdelhamid and El Bakkouri Jalila supervised the work, provided critical review for important intellectual content and gave final approval of the manuscript.

## Funding

This work received no specific grant from any funding agency in the public, commercial or non‐profit sectors.

## Disclosure

All authors read and approved the final version of this manuscript. All clinical content, data, interpretation and conclusions are the sole responsibility of the authors.

## Consent

The consent of the patient has been obtained to use the data and publish this article.

## Conflicts of Interest

The authors declare no conflicts of interest.

## Data Availability

The data that support the findings of this study are available upon request from the corresponding author. The data are not publicly available due to privacy or ethical restrictions.
